# Markedly elevated procalcitonin in early postoperative period in pediatric open heart surgery: a prospective cohort study

**DOI:** 10.1186/2052-0492-2-38

**Published:** 2014-06-20

**Authors:** Etsuko Minami, Shoji Ito, Takeshi Sugiura, Yoshihito Fujita, Hiroshi Sasano, Kazuya Sobue

**Affiliations:** Department of Anesthesiology and Medical Crisis Management, Nagoya City University Graduate School of Medical Sciences, Nagoya, Aichi, 467-8601 Japan; Department of Anesthesiology, Nagoya City East Medical Center, Nagoya, Aichi, 464-8547 Japan

**Keywords:** Procalcitonin, Cardiac surgery, Pediatric

## Abstract

**Background:**

We encountered markedly elevated procalcitonin (PCT) among pediatric patients during the early postoperative period of open heart surgery. The purpose of this study is to investigate what factors are associated with the PCT elevation.

**Methods:**

Fifty-two pediatric patients undergoing cardiac surgery with cardiopulmonary bypass (CPB) were enrolled. Plasma PCT, aspartate aminotransferase/alanine aminotransferase (AST/ALT), creatinine, lactate, and C-reactive protein (CRP) were measured on admission to ICU and during the postoperative period. The patients were categorized into high (group H) and low (group L) groups according to their peak PCT levels. Aorta cross-clamp (ACC), CPB time, ICU stay, mechanical ventilation period, peak AST/ALT, creatinine, lactate, and CRP levels were compared.

**Results:**

ACC and CPB times, ICU stay period, and mechanical ventilation period were significantly longer in group H compared with group L (118.7 ± 51.6 vs. 49.4 ± 43.5 min, 244.5 ± 65.7 vs. 122.9 ± 63.0 min, 7.9 ± 4.6 vs. 4.0 ± 4.5 days, and 6.3 ± 4.1 vs. 2.9 ± 4.2 days, respectively; *p* < 0.01). Peak AST and creatinine were significantly higher in group H compared with group L (999.0 ± 1,990.3 vs. 88.3 ± 43.0 U/l and 0.84 ± 0.77 vs. 0.41 ± 0.17 mg/dl, respectively; *p* < 0.05).

**Conclusions:**

ACC and CPB time-related perioperative stress is associated with elevated PCT; an association between ICU stay and mechanical ventilation period, liver enzymes, and creatinine levels was observed. PCT may be a good predictor of postoperative severity and organ dysfunction.

## Background

Procalcitonin (PCT) is a polypeptide composed of 116 amino acids. It is the precursor of calcitonin, which is involved in the metabolism of calcium. Under normal metabolic conditions, PCT is secreted by thyroid C cells. Although PCT has recently been advocated as a specific biomarker of severe infection, PCT in this context is supposed to have an extrathyroid origin and a different amino acid structure [1, 2]. Furthermore, the production of PCT is said to be associated with inflammatory cytokines such as tumor necrosis factor (TNF)-α [3]; it is also considered to be increased in not only infection but also systemic inflammatory response syndrome (SIRS).

We encountered a few cases of markedly elevated PCT levels among pediatric patients during the early postoperative period after aseptic cardiac surgery. We found several reports investigating changes in PCT levels during postoperative period in adult cases [4–6], but few reports in pediatric cases.

Therefore, in this study, we investigated what factors could be associated with marked elevation in PCT in the early postoperative period in pediatric cases.

## Methods

This study was approved by the ethical committee of Nagoya City University Hospital, and an agreement for participation in this study was obtained from each patient's family. Fifty-five pediatric patients, who were admitted to the ICU after open heart surgery with cardiopulmonary bypass (CPB), were enrolled. Three patients were excluded because of death during the early postoperative period. None of the patients had any clinical sign of infection on routine preoperative check. General anesthesia was maintained with large-dose fentanyl/midazolam in an oxygen-air mixture supplemented with sevoflurane and chlorpromazine. The method of CPB was almost identical in all patients. Methylprednisolone (50 mg/kg) was administered at the beginning of CPB.

Each patient's PCT level was measured on admission to the ICU and on postoperative days (PODs) 1, 2, 4, and 6. If the patient was discharged from the ICU before POD 6, the subsequent measurements of PCT were discontinued. PCT was measured by immunoluminometric assay (LUMI test PCT, WAKO, Osaka, Japan). Moreover, serum aspartate aminotransferase (AST), alanine aminotransferase (ALT), creatinine (CRE), C-reactive protein (CRP), and lactate (Lac) levels were also measured daily during the ICU stay.

Fifty-two patients were divided into two groups based on their peak PCT levels during the study period: PCT levels ≥5 ng/ml were categorized as group H and PCT levels <5 ng/ml were categorized as group L. We compared the following factors between the two groups: aorta cross-clamp (ACC) time, CPB time, ICU stay, mechanical ventilation period, and peak AST/ALT/CRE/Lac levels. Furthermore, we analyzed changes in PCT and CRP levels during the study. SPSS software ver. 11.0.1 J (IBM SPSS Inc., Chicago, IL, USA) was used for statistical analysis. For comparison of the patient backgrounds, Student's *t* test or a chi-square test was used. Measured parameters were compared with an unpaired *t* test, and changes in PCT and CRP levels over time were compared by two-way repeated measure ANOVA. Significance level was set at *p* < 0.05.

## Results

**Figure 1:**
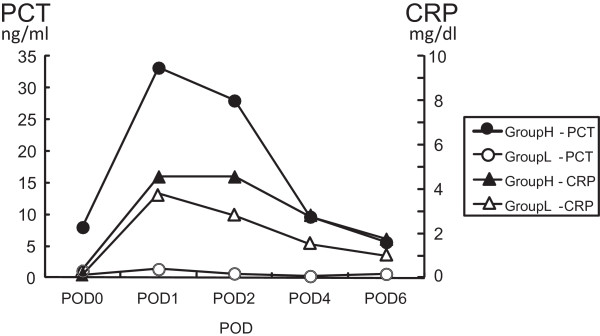
**The changes over time in PCT and CRP until POD6.** The mean values of PCT in group H became peak in the POD 1. There were not any significant differences between the two group's CRP levels in any days.

The preoperative characteristics of both groups are shown in Table 1. Twenty-three patients were classified as group H and 29 as group L. Their sex, age, and weight were not significantly different. The peak PCT level, shown as mean ± SD, in group H was 35.9 ± 45.8 ng/ml, while that in group L was 1.1 ± 1.4 ng/ml. Table 2 shows the surgical procedures performed in both groups. More complicated operations, such as the Fallot radical operation and the TCPC (total cavopulmonary bypass), were performed among group H patients, whereas simpler operations, such as closure of atrial and ventricular septal defects, were performed among group L patients. The ACC time, CPB time, ICU stay, and mechanical ventilation period were significantly longer among group H compared with group L patients. Although peak AST and CRE levels in group H were significantly higher than those in group L, peak ALT and Lac levels were not significantly different between the two groups (Table 3). Figure 1 shows the changes over time in the average PCT and CRP levels for both groups until POD 6. The average PCT levels among group H patients formed a very high peak on POD 1 and in subsequent PODs returned to the levels of POD 0. Furthermore, they were significantly higher compared with those in group L on all PODs. The CRP level was not significantly different among the two groups on any POD.

**Table 1:** **Characteristics of the patients**

Characteristic	Group H	Group L	***p***value
Male, female	14, 9	12, 17	NS
Age (months)	28.6 ± 19.9	39.2 ± 38.4	0.204
Weight (kg)	9.92 ± 3.93	12.0 ± 7.20	0.200
Peak PCT (ng/ml)	35.9 ± 45.8	1.14 ± 1.40	0.001

*NS* not significant.

**Table 2:** **Operative procedure**

	Group H	Group L
Fallot radical operation	6	0
TCPC	4	0
Bidirectional Glenn operation	3	2
DORV radical operation	3	0
VSD closure	2	6
ASD closure	1	13
Norwood operation	1	1
Others	3	7
Total	23	29

**Table 3:** **The comparison of each parameter between both groups**

	Group H	Group L	***p***value
ACC time (min)	118.7 ± 51.6	49.4 ± 43.5	<0.001
CPB time (min)	244.5 ± 65.7	122.9 ± 63.0	<0.001
ICU stay (days)	7.9 ± 4.6	4.0 ± 4.5	0.003
Mechanical ventilation (days)	6.3 ± 4.1	2.9 ± 4.2	0.005
AST (U/l)	999.0 ± 1,990.3	88.3 ± 43.0	0.039
ALT (U/l)	262.4 ± 662.8	15.3 ± 3.3	0.070
Creatinine (mg/dl)	0.84 ± 0.77	0.41 ± 0.17	0.014
Lactate (mg/dl)	34.8 ± 20.6	29.1 ± 24.4	0.380

## Discussion

The purpose of this study is to investigate the factors associated with the marked elevation of PCT observed during the early postoperative period after pediatric cardiac surgery with CPB was performed in aseptic condition. Longer ACC and CPB times and more complicated operations were observed among the patients with higher peak PCT levels. Franke et al. reported that PCT levels in patients after on-pump coronary artery bypass grafting were higher than those after off-pump coronary artery bypass surgery [7]. Hammer et al. compared PCT levels between groups of pediatric patients with longer and shorter ACC time after cardiac surgery with CPB and concluded that SIRS induced by cardiac surgery with CPB influenced PCT levels on the first day after surgery [8]. Similarly, Beghetti et al. reported that PCT levels were influenced by duration of CPB and ACC time [9]. In this study, we obtained comparable results, which indicate that the elevation in PCT levels could reflect intraoperative stress.

Among the pediatric patients with higher PCT levels, we observed prolonged ICU stay and mechanical ventilation period and elevated AST and CRE levels, which are indicative of liver or kidney dysfunction. Celebi et al. reported that PCT levels were increased excessively in SIRS accompanying organ failure after CPB in pediatric cardiac surgery and postoperative PCT levels correlated well with prolonged mechanical ventilation and hospitalization [10]. They also reported that the patients with SIRS and organ failure showed higher peak PCT levels. Beghetti et al. reported that PCT levels were influenced by the length of the mechanical ventilation period and ICU stay in children [9]. Given these findings, we believe that PCT levels during the early postoperative period are related to postoperative severity or organ failure and can be a predictor of these. ALT level was not significantly different between the two groups. However, there were three outliers in which ALT level exceeded 1,000 mg/dl in group H. When excluding these three outliers, ALT level was significantly higher in group H (*p* = 0.005). Hence, the use of PCT levels in postoperative management may be helpful for determining the suitability of therapeutic interventions during the early postoperative period.

Although changes in PCT levels over time among patients in group H formed a curve with a very high peak on POD 1 (which was clearly different from that in group L), no significant differences in changes in CRP levels were identified between the two groups. Generally, the CRP response is correlated with the magnitude of surgery, and it can be a marker of tissue damage [11]. There was no significant elevation of CRP levels among group H patients that would be indicative of severe surgical stress and tissue damage. We use steroids routinely during CPB. Although PCT is not supposed to be susceptible to steroids [12], CRP is. The elevation in CRP levels must thus have been masked due to the routine use of steroids during CPB. Aronen et al. found no statistical differences in concentrations between the complication and non-complication groups in their study of CRP kinetics in children undergoing open heart surgery [13]. Therefore, PCT would be a more suitable marker of intraoperative stress and a better predictor of postoperative severity and organ dysfunction than CRP.

Furthermore, in adult cases, several reports investigating changes in PCT levels showed similar results, but the absolute PCT values seem to be lower than those seen in pediatric cases [4, 5]. Although we can guess the causes, for example, pediatric patients have relatively larger surgical damage or metabolize PCT more slowly than adult patients, it remains unclear what is responsible for the differences in magnitude in PCT elevation between pediatric and adult patients.

Celebi et al. consistently observed high- or double-peak PCT curves in six infected patients after pediatric open cardiac surgery and claimed that these were suggestive of infection [10]. Although in our study there were no cases where PCT was elevated again by POD 6, if we had observed re-elevation of PCT in measurements taken after POD 6, it would have been necessary to take infection into consideration.

In our study, we divided the patients into two groups based on peak PCT levels higher and lower than 5 ng/ml. In cases of severe infection or sepsis, 2 ng/ml is used as a cutoff value. Macrina et al. claimed that a PCT concentration greater than 0.5 ng/ml was highly suggestive of a postoperative complication following CPB in adults [4]. However, because our pediatric cases had higher PCT levels (the highest value being 165 ng/ml), we thought it is suitable to use a cutoff value higher than that for severe infection and sepsis or for adult cases. Celebi et al. mentioned that the peak PCT of 5 had high sensitivity (100%) and specificity (95%) as the organ failure predictive cutoff values in pediatric open heart surgery [10].

## Conclusions

Prolonged ACC and CPB times, prolonged mechanical ventilation period and ICU stay, and higher serum AST and CRE levels were observed among patients with peak PCT levels >5 ng/ml. PCT levels during the early postoperative period can be an index of intraoperative stress and postoperative severity and a good predictor of organ dysfunction among pediatric patients after cardiac surgery with CPB.
